# Influence of capping chemotherapy prescriptions on efficacy and tolerability in medium and high-risk early-stage breast cancer

**DOI:** 10.1038/s41598-025-14279-3

**Published:** 2025-08-04

**Authors:** Wafa Bouleftour, Aurane Raphard, Fabien Tinquaut, Romain Rivoirard, Fabien Forges

**Affiliations:** 1 Medical Oncology Department, CHU Saint Etienne, Saint Etienne, France; 2 Pharmacy Department, CHU Saint Etienne, Saint Etienne, France; 3 Public Health and Medical Information Service, CHU Saint Etienne, Saint Etienne, France; 4 Medical Oncology Department, Private Loire Hospital, Saint-Etienne, France

**Keywords:** Capping, Breast cancer, Chemotherapy, Body surface area, Toxicities, Breast cancer, Cancer therapy

## Abstract

Capping body surface area (BSA) at 2.0 m^2^ is a common clinical practice. This empirical practice is intended to mitigate toxicities. In this context, the objective of this study was to investigate in curative situation whether capping chemotherapy prescriptions at 2.0 m^2^ had an influence on the efficacy and tolerance of treatment in patients diagnosed with early-stage breast cancer. Data from patients with a body surface area (BSA) greater than 2.0 m² who received treatment for medium and high-risk early-stage breast cancer, either in (neo)adjuvant settings, from January 1, 2010, to December 31, 2018, were examined. Patients were divided into four categories based on the percentage of chemotherapy capping throughout the treatment duration: [90–100]: the reference group, representing fully capped chemotherapy with capping exceeding 90%; [50–90[: capped chemotherapy ranging from 50 to 90%; [10–50[: capped chemotherapy between 10% and 50%; and [0–10[: representing non-capped chemotherapy. A total of 130 patients were included in the analysis, with a median age at diagnosis of 57 years (Interquartile range (IQR): 48–63) and a mean BSA of 2.07 m². Chemotherapy was provided as an adjuvant treatment to 86.9% of the participants. Depending on the capping group, the hematological toxicities were almost similar in all groups whereas non-hematological toxicities were slightly higher in the capped group between [10–50[. Similarly, chemotherapy dose reduction was also higher in capped group between [10–50[in comparison with other groups. A significant difference was observed in non-hematological toxicities of grade ≥ 2 between the reference group [90–100] and the capping group [10–50[(OR 3.59; 95% CI [1.26–10.22], *p* = 0.017). Prospective studies are needed to support the practice of capping, particularly in curative situations.

**Supplementary Information:**

The online version contains supplementary material available at 10.1038/s41598-025-14279-3.

## Introduction

Obesity is both a major public health issue and a well-established risk factor for chronic disease and cancer occurrence. Strong epidemiological evidence have linked obesity to increased cancer incidence and cancer-related mortality. Indeed, obesity was classified as the second most common preventable cause of cancer^1^. Recent studies have highlighted a complex interplay between obesity-related factors within the tumor and tumoral microenvironment through numerous complex metabolic pathways regulation such as p53, phosphoinositide 3-kinase (PI3K) and hypoxia-inducible factor 1α (HIF1α)^2^. In addition, obesity is also associated with worst prognosis, and its recognized as a predictor factor of cancer relapse^3^. The therapeutic management of obese patients with cancer remains a clinical challenge. Indeed, obesity alters pharmacokinetics of antineoplastic agents, by affecting tissue distribution and drug elimination^4^. To balance efficacy and toxicity, chemotherapy dosing is typically calculated based on body surface area (BSA), a parameter that was originally derived over a century ago from a population of individuals weighing between 25 and 90 kg—far from the current demographics of overweight and obese patients^5^. In clinical practice, alternative dosing strategies have emerged, including the use of ideal body weight, BSA capping (typically at 2.0 m²), and empirical dose reductions.

Capping BSA to 2.0 m² is a widely common clinical practice among healthcare professionals aimed at minimizing toxicities in patients with a BSA exceeding 2.0 m². Nevertheless, such medical practice is supported by neither scientific rationale nor clinical studies^6^. Indeed, such arbitrary dose limitations may result in significant underdoing, particularly in curative settings, where dose intensity is crucial for treatment success. Prior work by Bouleftour et al. demonstrated that this practice is highly variable and often depends more on physician preference (The same patient may undergo both capped and uncapped chemotherapy courses, based on the physician’s decision.) or treatment intent (curative vs. palliative) than on patient-.

Body Mass Index (BMI)^7^. The same study highlighted that capping was even frequently observed (> 60%) in patients with BSA over 2.0 m² and BMI less than 25, raising concerns about its rationale and consistency^7^. While few clinical trials have proposed capping, there are no oncological guidelines that endorse this clinical practice. The American Society of Clinical Oncology (ASCO) expert opinion updated in 2021, recommends not limiting chemotherapy doses in obese patients to avoid compromising clinical outcomes^8^. Furthermore, ASCO has reported that more than 40% of obese patients have received adjusted chemotherapy doses without sufficient justification^8^. While the impact of obesity on chemotherapy dosing has been broadly discussed, the specific consequences of BSA capping on treatment efficacy and toxicity in curative settings remain insufficiently studied. This is particularly relevant in medium and high-risk early-stage breast cancer, where therapeutic outcomes are closely tied to treatment intensity.

Therefore, the objective of this study was to evaluate whether capping chemotherapy prescriptions at 2.0 m² influences treatment efficacy and tolerance in obese patients with high-risk early-stage breast cancer receiving curative-intent chemotherapy. By focusing on real-world prescription patterns and treatment outcomes, this work seeks to clarify the clinical implications of BSA capping and contribute to evidence-based dosing practices.

## Results

### Patient’s characteristics

A total of 130 patients were included in the analysis, of which 126 were women (96.9%) and 4 were men (3.1%). The median age at diagnosis was 57 years (Interquartile range (IQR): 48–63). The average weight of the population was 100.2 kg, with a standard deviation of 10.8 kg. The median body mass index (BMI) was 35.8 kg/m², with values ranging from 32.7 to 39.3 kg/m². The mean BSA was 2.07 m². Notably, nearly 70% of the patients had comorbidities, with cardiovascular diseases being the most prevalent (43%), followed by diabetes (14%) and thyroid disorders (12.9%). Among those with cardiovascular conditions, hypertension was observed in 78.8% of patients, while rhythm disorders and thromboembolic events were present in 8.7% and 6.3% of patients, respectively. A prior history of cancer was observed in 15.4% of the patients with breast cancer accounting for 65% of these cases, affecting 13 patients. The vast majority of patient’s (80%) had infiltrating ductal carcinoma. Regarding SBR grading, which reflects the aggressiveness of the tumor, 51.5% of patients had grade 2 and 42.3% a grade 3. All clinical characteristics are detailed in Tables 1 and 2.

**Table 1:** Patient’s characteristics.

	All patients	Percentage of capping				*P*-value
		[0–10[	[10–50[	[50–90[	[90–100]	
No. of patients (%)	130 (100%)	13 (10%)	42 (32.3%)	50 (38.5%)	25 (19.2%)	
Women, N (%)	126 (96.9%)	13 (100%)	42 (100%)	48 (96%)	23 (92%)	0.274
Age (years), median [Q1, Q3]	57 [48–63]	53 [48–60]	53 [48–62]	59 [50–65]	55 [47–63]	0.288
Weight (kg), median [Q1, Q3]	99,5 [95–105]	98 [93–103]	98 [92,2-105]	99.5 [95-102.8]	102 [97–109]	
BMI (kg/m^2^), median [Q1, Q3]	35,8 [32.7–39.3]	36,6 [33.3–37.8]	35.5 [32.5–39.1]	36,1 [32.1–38.9]	37 [32.7–40.9]	0.762
Menopausal patient, N (%)	72 (55.4%)	7 (53.8%)	21 (50%)	32 (64%)	12 (48%)	0.616
Concurrent pathologies, N (%)	90 (69.2%)	10 (76.9%)	25 (59.5%)	38 (76%)	17 (68%)	0.506
Cardiovascular diseases, N (%)	80 (43%)	9 (52.9%)	18 (38.3%)	35 (44.9%)	18 (40.9%)	
Diabetes, N (%)	26 (14%)	1 (5.9%)	9 (19.1%)	10 (12.8%)	6 (13.6%)	
Previous cancer (including breast cancer), N (%)	20 (15.4%)	3 (23.1%)	10 (23.8%)	5 (10%)	2 (8%)	0.169
Previous chemotherapy, N (%)	10 (7.7%)	0 (0%)	5 (11.9%)	3 (6%)	2 (8%)	

**Table 2:** Cancer and chemotherapy characteristics.

		All patients	Group of capping				*P*-value
			[0–10[	[10–50[	[50–90[	[90–100]	
No. of patients (%)		130 (100%)	13 (10%)	42 (32.3%)	50 (38.5%)	25 (19.2%)	
Type of cancer	Infiltrating duct carcinoma	104 (80%)	10 (76.9%)	32 (76.2%)	40 (80%)	22 (88%)	0.705
	Infiltrating lobular carcinoma	21 (16.2%)	1 (7.7%)	8 (19%)	9 (18%)	3 (12%)	0.802
	Other carcinoma	5 (3.8%)	2 (15.4%)	2 (4.8%)	1 (2%)	0 (0%)	0.853
Grade SBR	1	6 (4.6%)	1 (7.7%)	2 (4.8%)	2 (4%)	1 (4%)	0.79
	2	67 (51.5%)	5 (38.5%)	21 (50%)	25 (50%)	16 (64%)	
	3	55 (42.3%)	7 (53.8%)	18 (42.9%)	22 (44%)	8 (32%)	
	MD	2 (1.5%)	0 (0%)	1 (2.4%)	1 (2%)	0 (0%)	
Metastasis status (M)	MX	16 (12.3%)	2 (15.4%)	5 (11.9%)	7 (14%)	2 (8%)	–
	M0	114 (87.6%)	11 (84.6%)	37 (88%)	43 (86%)	23 (92%)	
HER2 status	Positive	23 (17.7%)	3 (23.1%)	10 (23.8%)	7 (14%)	3 (12%)	0.472
	Negative	107 (82.3%)	10 (76.9%)	32 (76.2%)	43 (86%)	22 (88%)	
Estrogen receptors	Positive	96 (73.8%)	9 (69.2%)	27 (64.3%)	39 (78%)	21 (84%)	0.26
	Negative	34 (26.2%)	4 (30.8%)	15 (35.7%)	11 (22%)	4 (16%)	
Progesterone receptors	Positive	79 (60.8%)	10 (76.9%)	19 (45.2%)	30 (60%)	20 (80%)	0.023*
	Negative	51 (39.2%)	3 (23.1%)	23 (54.8%)	20 (40%)	5 (20%)	
Chemotherapy	Adjuvant	113 (86.9%)	13 (100%)	31 (73.8%)	46 (92%)	23 (92%)	0.028*
	Neoadjuvant	17 (13.1%)	0 (0%)	11 (26.2%)	4 (8%)	2 (8%)	
Number of cycle received, mean [± SD]		7(± 3.4)	6.5 (± 3.5)	8 (± 4)	6.5 (± 2.9)	6 (± 2.8)	0.034*

### Treatment characteristics, and prescriptions capping

Chemotherapy was administered as an adjuvant treatment to 86.9% of the patients (*n* = 113), while 13.1% (*n* = 17) received neoadjuvant chemotherapy.

A statistically significant difference was observed between the 4 groups regarding the chemotherapy setting (adjuvant vs. neoadjuvant) (*p* = 0.028). Among patients, 23 individuals has HER2 overexpression and received concomitant trastuzumab administration, representing 17.7% of the cohort.

In this cohort, 13 patients (10%) had prescriptions that were either uncapped or marginally capped. In contrast, the reference group consisted of 25 patients (19.2%) whose prescriptions were consistently capped. The remaining patients were categorized into two intermediate capping groups: 42 patients were categorized into the 10–50% capping range, while 50 patients were classified in the 50–90% capping range. Interestingly, the average number of chemotherapy cycles received by the [10–50] group was significantly higher than other groups (*p* = 0.034) (Table 2). Of all chemotherapy cures received by patients, capped and uncapped cures account for 50.5% and 49.5% respectively. For patients whose doses have been capped, the variation in dosage compared to the actual BSA-based dosage was calculated. The deviation ranges from 0 to 12.2%, with a median deviation of 1.2% [0.4; 2.7]. Excluding uncapped treatments, the median dose variation “lack of dosage” per patient was 2.5% [1.4-5].

### Toxicities occurrence and capping

Among the 130 patients studied, 68.5% (*n* = 89) experienced toxicities following the administration of chemotherapy. The analysis revealed that 77 patients (59.2%) encountered at least one non-hematologic toxicity of grade ≥ 2, while 29 patients (22.3%) reported at least one grade ≥ 3 hematologic toxicity. In total, 178 non-hematologic events of grade ≥ 2 were documented. The predominant events included nausea (*n* = 51), asthenia (*n* = 42), neurotoxicity (*n* = 22), diarrhea (*n* = 15), and hand/foot syndrome (*n* = 13). Hematologic toxicities of grade 3 and grade 4 represented 48.7% and 51.3%, respectively.

The analysis of the percentage of patients with at least one toxicity across all groups, showed that the dose reduction of chemotherapy was significant (*p* = 0.017). Regarding hematological toxicities, a slight trend was also observed in the occurrence of agranulocytosis (*p* = 0.071). No significant difference was observed in the occurrence of non-hematological toxicities except for alopecia (*p* = 0.002) (Table 3).

**Table 3:** Percentage of patients presenting at least one event according to the capping percentage.

	Total (*n* = 130)	0–10 (*n* = 13)	10–50 (*n* = 42)	50–90 (*n* = 50)	90–100 (*n* = 25)	*P* value
Patient with at least one toxicity		**7 (53.8)**	**34 (80.9)**	**36 (72)**	**16 (69.5)**	
Hematological toxicities Grade ≥ at 3						
Agranulocytosis	**5 (3.8)**	0 (0)	**3 (7.1)**	**2 (4)**	0 (0)	*p* = 0.071
Leucopenia	**4 (3.1)**	0 (0)	**2 (4.7)**	**1 (2)**	**1 (4)**	*p* = 0.56
Neutropenia	**21 (16.2)**	**2 (15.4)**	**9 (21.4)**	**7 (14)**	**3 (12)**	*P* = 0.79
Anemia	**3 (2.3)**	0 (0)	0 (0)	**2 (4)**	**1 (4)**	*p* = 0.33
Febrile aplasia	**7 (5.4)**	**2 (15.4)**	**1 (2.3)**	**3 (6)**	**1 (4)**	*p* = 0.32
Thrombopenia	**3 (2.3)**	0 (0)	0 (0)	**2 (4)**	**1 (4)**	*P* = 0.32
Non-hematological toxicities Grade ≥ at 2						
Asthenia	**34 (26.2)**	**2 (15.4)**	**10 (23.8)**	**14 (28)**	**8 (32)**	*p* = 0.50
Stomatitis	**11 (8.5)**	**2 (15.4)**	**4 (9.5)**	**3 (6)**	**2 (8)**	*p* = 0.73
Nausea	**34 (26.2)**	**6 (46.1)**	**12 (28.5)**	**11 (22)**	**5 (20)**	*P* = 0.28
Nail toxicity	**4 (3.1)**	**1 (7.7)**	**3 (7.1)**	0 (0)	0 (0)	*p* = 0.98
Skin toxicity	**7 (5.4)**	0 (0)	**4 (9.5)**	**3 (6)**	0 (0)	*p* = 0.31
Paresthesia	**4 (3.1)**	0 (0)	**1 (2.3)**	**3 (6)**	0 (0)	*p* = 0.44
Neuropathy	**10 (7.7)**	0 (0)	**5 (11.9)**	**3 (6)**	**2 (8)**	*p* = 0.56
Alopecia	**2 (1.5)**	0 (0)	0 (0)	**2 (4)**	0 (0)	*p* = 0.002
Hand-foot syndrome	**9 (6.9)**	0 (0)	**4 (9.5)**	**2 (4)**	**3 (12)**	*p* = 0.44
Cephalalgia	**1 (0.8)**	0 (0)	0 (0)	**1 (2)**	0 (0)	*p* = 1
Anorexia	**2 (1.5)**	0 (0)	0 (0)	**1 (2)**	**1 (4)**	*p* = 0.36
Hot flash	**1 (0.8)**	0 (0)	**1 (2.3)**	0 (0)	0 (0)	*p* = 1
Vomiting	**2 (1.5)**	0 (0)	**1 (2.3)**	0 (0)	**1 (4)**	*p* = 0.36
Constipation	**1 (0.8)**	0 (0)	**1 (2.3)**	0 (0)	0 (0)	*p* = 1
Pain	**1 (0.5)**	0 (0)	0 (0)	0 (0)	**1 (4)**	*p* = 1
Diarrhea	**13 (10)**	**1 (7.7)**	**6 (14.2)**	**5 (10)**	**1 (4)**	*p* = 0.69
**Chemotherapy dose cancellation**	**7 (5.4)**	0 (0)	**2 (4.7)**	**5 (10)**	0 (0)	0.18
**Chemotherapy dose reduction**	**35 (26.9)**	0 (0)	**17 (40.4)**	**10 (20)**	**8 (32)**	*p* = 0.017
**Chemotherapy delay**	**4 (3.1)**	0 (0)	**2 (4.7)**	**1 (2)**	**1 (4)**	*p* = 0.64
**Hospitalization**	**18 (13.8)**	**3 (23.1)**	**4 (9.5)**	**8 (16)**	**3 (12)**	*p* = 0.39

### Progression free survival and overall survival

The median progression-free survival was not reached (*p* = 0.3). The median follow-up (Q1-Q3) was: 5.28 (3.84–7.1). The likelihood of event-free survival at the 2-year was 92.4%, corresponding to 11 events (either progression or death). At 4 years, the event-free survival probability decreased to 85.9%, with 18 events recorded, and at 6 years, it further declined to 78%, with a total of 24 events. The median overall survival was also not reached. The probability of overall survival at 2 years was 98.8%, with 4 fatalities recorded since the start of the study. At 4 years, the probability of overall survival was 94.1%, with 9 deaths, and at 6 years 87.5%, with 13 deaths (Supplementary materials).

### Logistic regression analysis

Regarding logistic regression analysis, patients whose capping percentage throughout their treatment ranged from 90 to 100% was considered as the reference group.

Univariate analysis revealed only a statistically significant difference in the occurrence of all toxicities between the reference group [90–100] and the “moderately capped” group [10–50[(OR 3.34; 95% CI [1.11–10.06]; *p* = 0.032). This result indicated that patients in the “moderately capped” group are 3.34 more likely to experience toxicity than the reference group [90–100].

Regarding non-hematological toxicities of grade ≥ 2, a significant difference was observed between the reference group [90–100] and the “moderately capped” group [10–50[(OR 3.59; 95% CI [1.26–10.22], *p* = 0.017). This results indicated that the “moderately capped” group are 3.59 more likely to experience non-hematological toxicities than the reference group [90–100].

Conversely, no statistically significant difference was found regarding the occurrence of grade ≥ 3 hematological toxicities between the groups (*p* > 0.05). Additionally, there was no significant difference noted among the various chemotherapy dose variation groups concerning the presence of toxicities (*p* = 0.517) (Table 4).

In contrast to univariate analysis, the Multivariable analysis showed no statistical difference in the occurrence of all toxicities between the reference group [90–100] and the “moderately capped” group (OR 2.15; 95% CI [0.67–6.91], *p* = 0.197).

**Table 4:** Univariate and multivariable logistic regression regarding toxicity occurrence and the percentage of capping.

Variable	Univariate			Multivariable		
	Capping groups	OR (95% CI)	*p*		Adjusted OR (95% CI)	*p*
All toxicities	**Reference group [90–100]**			**Reference group [90–100]**		
	[0–10[	0.92 (0.24–3.52)	0.899	[0–10[	0.92 (0.23–3.72)	0.909
	[10–50[	3.34 (1.11–10.06)	**0.032**	[10–50[	2.15 (0.67–6.91)	0.197
	[50–90[	1.67 (0.62–4.49)	0.309	[50–90[	1.34 (0.48–3.78)	0.576
Hematologic toxicities	**Ref=[90–100]**					
	[0–10[	0.95 (0.15–6.06)	0.961			
	[10–50[	2.1 (0.59–7.41)	0.249			
	[50–90[	1.48 (0.42–5.42)	0.542			
Non-hematologic toxicities	**Ref=[90–100]**					
	[0–10[	1.48 (0,39-5.71)	0.565			
	[10–50[	3.59 (1.26–10.22)	**0.017**			
	[50–90[	1.62 (0.62–4.26)	0.328			

All toxicities represent the hematological toxicities grade ≥ 3 and the non-hematological toxicities grade ≥ 2 according to the CTCAE v5.0 criteria. Hematological toxicities were defined as a grade ≥ 3 and the non-hematological toxicities were defined as a grade ≥ 2 OR: odds ratio.

Regarding Progression-Free Survival (PFS), no notable differences were found when comparing the reference group to the [0–10[ and [50–90[ capping groups. However, a significant difference was identified between the reference group and the [10–50[ capping group (HR 5.02, 95% CI 1.12–22.53, *p* = 0.035). This result indicate that, [10–50[ capping group had 5.02 times greater risk of progression compared to the reference group (Table 5).

In the univariate analysis, patients who received neoadjuvant treatment had nearly 3-fold higher risk of progression than those receiving adjuvant treatment (HR = 2.93; 95% CI [1.17–7.37], *p* = 0.118). This risk decreased to HR = 2.19 (95% CI [0.82–5.87], *p* = 0.139) ([0.82–5.87], *p* = 0.139 in multivariable analysis, However, these results are not statistically significant and must should be interpreted with caution (Table 5).

**Table 5:** Univariate logistic regression regarding progression free survival (PFS).

Variable	Univariate			Multivariable		
	Capping groups	OR (95% CI)	*p*		Adjusted OR (95% CI)	*p*
Capping group	**Reference group [90–100]**			**Reference group [90–100]**		
	[0–10[	2.61 (0.43–15.67)	0.294	[0–10[	2.59 (0.43–15.73)	0.302
	[10–50[	5.02 (1.12–22.53)	**0.035**	[10–50[	3.79 (0.81–17.74)	0.09
	[50–90[	3.33 (0.75–14.76)	0.113	[50–90[	3.05 (0.68–13.68)	0.145
Chemotherapy	Neoadjuvant vs. adjuvant	2.93 (1.17–7.37)	0.118		2.19 (0.82–5.87)	0.139

*HR* Hazard ratio.

## Discussion

The global rise in obesity and overweight conditions has not yet led to a validated approach for modifying anticancer drug dosages in these populations. Nevertheless, practice of chemotherapy dose reduction by BSA capping in overweight and obese patients is frequently used, particularly in medical oncology^7^. In this study, 57.7% of patients had their treatment prescriptions capped at 50% or more. In the context of early-stage breast cancers, which are typically curative, doses should theoretically not be limited—barring exceptional circumstances such as advanced age, poor performance status, or co-morbidities—to avoid compromising the effectiveness of the treatment, even if this necessitates subsequent management of any resulting toxicities. The selection of a population undergoing treatment for medium and high risk early breast cancer with a BSA > 2.0 m² allowed us to describe a homogeneous population in terms of anthropometric *characteristics* (women, BMI > 25).

CALGB 49,907 was a phase 3 trial that investigated actual-weight-based adjuvant chemotherapy dosing in women aged over 65 with early-stage breast cancer. The analysis of toxicities showed that the rates of toxicity were 30.5% in overweight patients and 22% in obese patients^9^. In our research, the incidence of grade ≥ 3 hematological toxicities in a population of patients aged over 65 years was found to be 22.3%, which aligns with the findings of Morrison’s study^9^. The rates of grade ≥ 3 non-haematological toxicities in overweight and obese older patients reported by Morrison et al. were 36% and 41.4%, respectively. However, our study recorded a significantly lower rate of these toxicities at only 8.5%, of grade ≥ 3 non-haematological toxicities in patients aged over 65 year^9^. These discrepancies, especially concerning non-haematological toxicities, indicate that older patients may experience a higher frequency of adverse effects compared to younger patients.

For haematological toxicities, 34 grade ≥ 3 events occurred in our cohort of 130 patients, corresponding to a rate of 26.2%. This rate is lower than those reported in the literature^9,10^. This discrepancy may be attributed to the increased use of hematopoietic growth factors for primary prevention, especially following the occurrences of docetaxel toxicity in 2017. Notably, 64.6% of the patients in our study, totaling 84 patients, received hematopoietic growth factors.

In this retrospective cohort, the practice of capping did not significantly reduce toxicities in overweight or obese patients. These results are consistent with those observed in studies on breast cancer treated with adjuvant chemotherapy^9,11,12^. In addition, the analysis of a retrospective study involving 662 patients indicated that overweight breast cancer patients who received adjuvant chemotherapy dosed according to their actual weight do not face an increased risk of experiencing myelosuppressive events. The study concluded that patients should be administered the full dose of chemotherapy based on BSA^13^. Similarly, Poikonen et al., retrospective study concluded that adjuvant postoperative cyclophosphamide, methotrexate and fluorouracil administration used for breast cancer treatment, should not be reduced because of obesity^14^. In contrast, GAIN retrospective analysis, which included data of 555 patients with a BMI over than 30, observed an increased rate of global toxicities (especially high grade of hematological toxicities)^10^.

In this retrospective study, unexpectedly, patients of moderately capped [10–50[ group, presented a significant increase in non-hematological toxicities. This may be because this group received a higher number of chemotherapy cycle compared to other group (8 vs. 6 in the reference group) (Table 2). Gain retrospective study, showed significant differences in non-hematological toxicities between the unadjusted and adjusted groups (Diarrhea 18.5% vs. 27.2, *p* = 0.0033; renal and urinary disorders 10.4% vs. 17.5%, *p* = 0.031; dizziness 4.6% vs. 11, *p* = 0.016; and hot flushes 2.9% vs. 0.3%,*p* = 0.013)^10^. In contrast, several studies showed that capping chemotherapy doses in obese patients does not significantly reduce non-hematological toxicities^8^. Thus, prospective studies are needed to elucidate these discrepancies. This study did not show any effect of capping on treatment efficacy. In the context of neoadjuvant breast cancer treatment, Farr et al. showed the interest of using actual body weight in improving histological response and PFS^15^. Unexpectedly, the results showed a trend towards a lower risk of progression in the capped patient groups. The observed phenomenon can be attributed to the higher percentage of patients receiving neoadjuvant chemotherapy within the capping group [10–50[ (26.2%, *n* = 11) in contrast to the capping group [90–100] (8%, *n* = 2), whose tumors are more aggressive and have a poorer prognosis. In addition, the difference in toxicity can be explained in the same way because neoadjuvant treatments are more aggressive than adjuvant treatments. Finally, the dose variations induced by capping are in most cases low and could be considered clinically negligible but this does not justify this practice.

Regarding PFS, while the Kaplan-Meier method did not reach statistical significance (*p* = 0.3; supplementary material), the univariate model identified a significant increase in progression risk for the [10–50[ capping group compared to the reference group (HR = 5.02, *p* = 0.035). This indicates that pairwise differences may exist despite the absence of a global survival difference, highlighting the greater sensitivity of the Cox model for detecting specific contrasts.”

The primary limitation of this study is the insufficient statistical power resulting from the relatively small patient cohort, which led to a bias-inducing distribution of some criteria of interest. Additionally, variability in physician practices for the same patient contributed to the inability to establish clearly defined “capped” and “non-capped” patient groups. Indeed, during each chemotherapy cycle, patients might be assessed by different physicians as part of their treatment plan. The decision to limit each chemotherapy prescription was affected by healthcare practices, leading to inconsistent application of capping for patients. The design of the chemotherapy prescription software and the absence of consideration regarding capping may account for this observed lack of homogeneity. In addition, the statistical interpretation of the significant association between capping and non-hematological toxicities was not performed, given the small number of samples in each group, as well as the heterogeneity of clinical characteristics (e.g.: 21 different chemotherapy regimens and cancer stages).

In conclusion, this study raises important questions about the potential benefits and risks of chemotherapy dose capping, particularly in curative cancer treatment settings. While dose capping may seem protective in theory, current high-level evidence does not support routine capping in curative-intent treatment. In addition, sarcopenic obesity challenges the conventional rationale for chemotherapy capping. Indeed, sarcopenia is an independent predictor of chemotherapy toxicity, including non-hematologic toxicities^16,17^. Thus, the identification of sarcopenic individuals who may require personalized dosing strategies or closer toxicity monitoring have to be also assessed.

In addition, the proliferation of fixed-dose prescriptions, such as monoclonal antibodies or dose banding, highlights the necessity for more prospective clinical trials and pharmacological research to resolve the ambiguities surrounding cancer treatment in obese patients. Such studies would facilitate the establishment of a definitive consensus regarding the practice of capping.

## Materials and methods

This retrospective study was carried out in a French anticancer institute. All data of patients with BSA > 2.0 m^2^ and treated for locally advanced breast cancer (adjuvant or neo-adjuvant settings) between January 1st 2010 and December 31th 2018 were analyzed. This study was approved by Saint Etienne local ethics committee (Ref: IRBN1422020/CHUSTE) and was conducted according to the relevant guidelines and regulations. In addition, that informed consent was obtained from all subjects.

### Patient selection

**Fig. 1:**
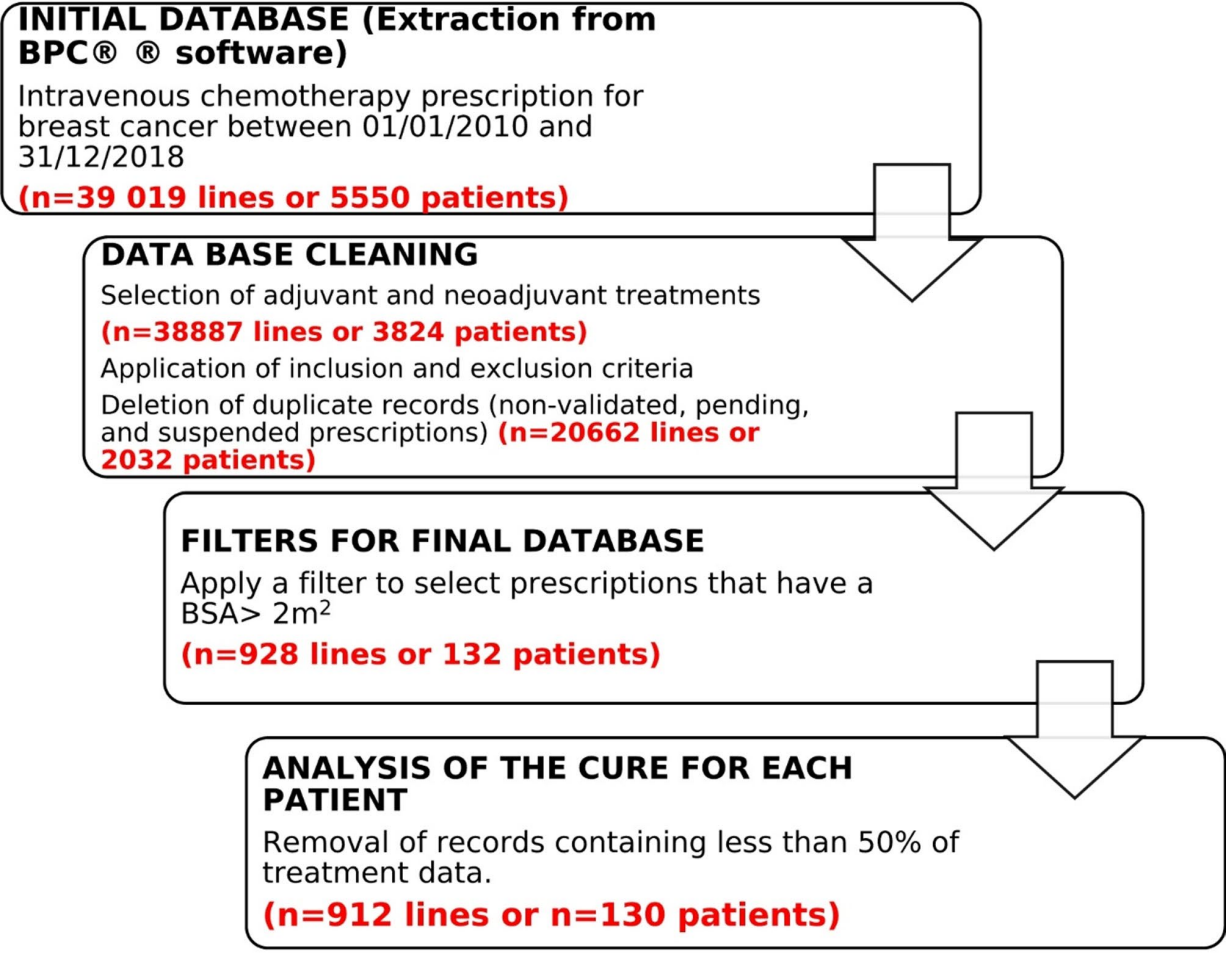
Schematic plan of the methodology of data collection.

The prescriptions were extracted from the prescription software BPC^®^. A total of 39,019 computerized prescriptions were extracted in EXCEL format. Subsequently, 130 patients were then selected (Fig. 1). Patients with BSA > 2.0m^2^ and with histologically confirmed medium/high-risk early-stage breast cancer, treated by chemotherapy in the (neo)adjuvant setting between January 2010 and December 2018, were included. Patients with synchronous metastatic breast cancer, confirmed by imagery and/or by biopsy, were excluded.

The analysis excluded prescriptions for patients enrolled in a clinical trial because of the regular requirements for antineoplastic dose calculations. Systematic dose capping for example at 800 mg for carboplatin were not considered as a dose capping. Furthermore, any records that were incomplete, specifically those with less than 50% of the data for all treatments available (patients partially treated at the institute), were also excluded (Fig. 1).

The BPC^®^ software applies the Dubois and Dubois formula to compute BSA. In instances where the BSA exceeds 2.0 m², the software prompts physicians to select whether to impose a capping on the prescription.

### Data analysis

Data were collected from medical records, based on hospital reports and biological analysis. These data were then entered in an eCRF. Age calculation was based on the interval between the date of the first chemotherapy prescription and the patient’s date of birth. The BMI was calculated from the height and weight noted at the time of the first chemotherapy prescription, following the formula: weight (kg) / height² (m²). The adverse events were collected from patient’s medical file and then interpreted in accordance with the Common Terminology Criteria for Adverse Events (CTCAE V 5.0). Throughout their treatment regimen, patients could be evaluated by various physicians during each chemotherapy cycle. The approach to cap each chemotherapy prescription was influenced by medical practices and the functionality of the prescription software BPC^®^, resulting in a variable application of capping for patients. Indeed, at each chemotherapy cycle, prescriptions were validated by different physicians, resulting in inconsistent application of chemotherapy dose capping. This variability could be explained by the absence of clear guidelines about capping and by the chemotherapy prescription software. Consequently, it was not feasible to categorize the patients into two distinct groups of “capped” and “uncapped.” Thus, to address this issue, the capping criteria was therefore characterized as a quantitative variable rather than a qualitative one, ranging from 0% (indicating a patient who has never been capped) to 100% (indicating a patient who has consistently been capped). The statistical analysis categorized the data into four groups based on the percentage of capping throughout the entire treatment.

- [90–100]: The capped population consists of patients who have undergone fully capped chemotherapy, as well as those whose chemotherapy capping exceeds 90%, serving as the reference group.
- [50–90[: patients who received capped chemotherapy between 50 and 89%.
- [10–50[: patients who received capped chemotherapy between 10 and 49%.
- [0–10[: The Non-capped population (patients who received uncapped chemotherapy (doses based on real weight and body surface area), along with those whose chemotherapy capping does not surpass 10%.

For each patient, the “a lack of dosage” was estimated by the difference between the dose calculated with the actual BSA and the dose calculated after capping the BSA at 2.0 m². A threshold of 3% was determined arbitrarily, representing the maximum dose deviation tolerated in practice at our institute. Thus any dose deviation exceeding 3% was considered significant.

### Statistical analysis

All variables collected were described using the following methods: average (standard deviation), median IQR, for quantitative variables; size (percentage) for qualitative variables. The comparability of groups was assessed using Fisher’s exact tests for qualitative variables and Wilcoxon tests for quantitative variables. Survival times were defined as the duration from the initial treatment date to the occurrence of the event. In cases of relapse or disease progression, the date of imaging that confirms the occurrence was used. Survival outcomes were analyzed using Kaplan-Meier. All comparisons were performed using the Log-Rank test. For each comparison, a Hazard Ratio (HR) along with its 95% confidence interval was presented, derived from a Cox proportional hazards model.

Variables with a p-value below a threshold of 0.2 in univariate analysis were retained as candidates for multivariate analysis. Indeed, these variables were: gender, age, BMI, menopausal status, tobacco and alcohol use, concomitant pathology (yes/no), cancer history (yes/no), radiotherapy history, chemotherapy history, metastasis status.

The effect of capping on toxicity was evaluated using Fisher-exact test. The multivariable analysis was performed with logistic regression, the alpha risk is set at 0.05 and the analyses was performed with the R language and environment for statistical computing version 3.5.2, with the package “lme4” version 1.1–19.

## Supplementary Information

Below is the link to the electronic supplementary material.

Supplementary Material 1

## Data Availability

“The datasets used and/or analysed during the current study available from the corresponding author on reasonable request.”

## References

[1] Lazarus, E. & Bays, H. E. Cancer and obesity: an obesity medicine association (OMA) clinical practice statement (CPS) 2022. *Obes. Pillars*. **3**, 100026 (2022). 37990728 10.1016/j.obpill.2022.100026 PMC10661911

[2] Brown, K. A. Metabolic pathways in obesity-related breast cancer. *Nat. Rev. Endocrinol.* **17**, 350–363 (2021). 33927368 10.1038/s41574-021-00487-0 PMC10410950

[3] Lohmann, A. E. et al. Association of obesity with breast cancer outcome in relation to cancer subtypes: a meta-analysis. *J. Natl. Cancer Inst.* **113**, 1465–1475 (2021). 33620467 10.1093/jnci/djab023 PMC8562970

[4] Thimotheo Batista, J. P. et al. Chemotherapy and anticancer drugs adjustment in obesity: a narrative review. *Curr. Med. Chem.* **30**, 1003–1028 (2023). 35946096 10.2174/0929867329666220806140204

[5] Du Bois, D. & Du Bois, E. F. A formula to estimate the approximate surface area if height and weight be known. 1916. *Nutrition* **5**, 303–311 (1989). 2520314

[6] Bouleftour, W. et al. Obesity and chemotherapy administration: between empiric and mathematic method review. *Acta Oncol.* **58**, 880–887 (2019). 30907190 10.1080/0284186X.2019.1585942

[7] Bouleftour, W. et al. Body surface area capping May not improve cytotoxic drugs tolerance. *Sci. Rep.* **11**, 2431 (2021). 33510207 10.1038/s41598-021-81792-6 PMC7843991

[8] Griggs, J. J. et al. Appropriate chemotherapy dosing for obese adult patients with cancer: American society of clinical oncology clinical practice guideline. *J. Clin. Oncol.* **30**, 1553–1561 (2012). 22473167 10.1200/JCO.2011.39.9436

[9] Morrison, V. A. et al. The impact of actual body weight-based chemotherapy dosing and body size on adverse events and outcome in older patients with breast cancer: results from cancer and leukemia group B (CALGB) trial 49907 (Alliance A151436). *J. Geriatr. Oncol.* **9**, 228–234 (2018). 29233548 10.1016/j.jgo.2017.11.007 PMC5936657

[10] Furlanetto, J. et al. Higher rate of severe toxicities in obese patients receiving dose-dense (dd) chemotherapy according to unadjusted body surface area: results of the prospectively randomized GAIN study. *Ann. Oncol.* **27**, 2053–2059 (2016). 27502721 10.1093/annonc/mdw315

[11] Lote, H. et al. Febrile neutropenia rates according to body mass index and dose capping in women receiving chemotherapy for early breast cancer. *Clin. Oncol. (R Coll. Radiol)*. **28**, 597–603 (2016). 26936608 10.1016/j.clon.2016.02.003

[12] Carroll, J. P. et al. Toxicity and tolerability of adjuvant breast cancer chemotherapy in obese women. *Med. Oncol.* **31**, 881 (2014). 24549982 10.1007/s12032-014-0881-z

[13] Jenkins, P., Elyan, S. & Freeman, S. Obesity is not associated with increased myelosuppression in patients receiving chemotherapy for breast cancer. *Eur. J. Cancer*. **43**, 544–548 (2007). 17169553 10.1016/j.ejca.2006.10.013

[14] Poikonen, P., Blomqvist, C. & Joensuu, H. Effect of obesity on the leukocyte nadir in women treated with adjuvant cyclophosphamide, methotrexate, and fluorouracil dosed according to body surface area. *Acta Oncol.* **40**, 67–71 (2001). 11321664 10.1080/028418601750071082

[15] Farr, A. et al. The effect of obesity on pathological complete response and survival in breast cancer patients receiving uncapped doses of neoadjuvant anthracycline-taxane-based chemotherapy. *Breast* **33**, 153–158 (2017). 28395233 10.1016/j.breast.2017.04.001

[16] Carneiro, I. P., Mazurak, V. C. & Prado, C. M. Clinical implications of sarcopenic obesity in cancer. *Curr. Oncol. Rep.* **18**, 62 (2016). 27541923 10.1007/s11912-016-0546-5

[17] Baracos, V. E. & Arribas, L. Sarcopenic obesity: hidden muscle wasting and its impact for survival and complications of cancer therapy. *Ann. Oncol.* **29**, ii1–ii9 (2018). 29506228 10.1093/annonc/mdx810

