# Correction: Influence of capping chemotherapy prescriptions on efficacy and tolerability in medium and high-risk early-stage breast cancer

**DOI:** 10.1038/s41598-025-33450-4

**Published:** 2025-12-24

**Authors:** Wafa Bouleftour, Aurane Raphard, Fabien Tinquaut, Romain Rivoirard, Fabien Forges

**Affiliations:** 1 Medical Oncology Department, CHU Saint Etienne, Saint Etienne, France; 2 Pharmacy Department, CHU Saint Etienne, Saint Etienne, France; 3 Public Health and Medical Information Service, CHU Saint Etienne, Saint Etienne, France; 4 Medical Oncology Department, Private Loire Hospital, Saint-Etienne, France

Correction to: *Scientific Reports* 10.1038/s41598-025-14279-3, published online 04 August 2025.

The original version of this Article contained an error in the name of authors Wafa Bouleftour, Aurane Raphard, Fabien Tinquaut, Romain Rivoirard and Fabien Forges, which were incorrectly given as Bouleftour Wafa, Raphard Aurane, Tinquaut Fabien, Rivoirard Romain and Forges Fabien.

The original Article has been corrected.

