# Supplementary material for: Influence of capping chemotherapy prescriptions on efficacy and tolerability in medium and high-risk early-stage breast cancer
Source: Sci Rep. 2025 Aug 4;15:28415. doi: 10.1038/s41598-025-14279-3 (PMC12322036; doi:10.1038/s41598-025-14279-3)
Supplement: Supplementary file 1 — Supplementary Material 1 [file 41598_2025_14279_MOESM1_ESM.docx]

**Supplementary materials:**


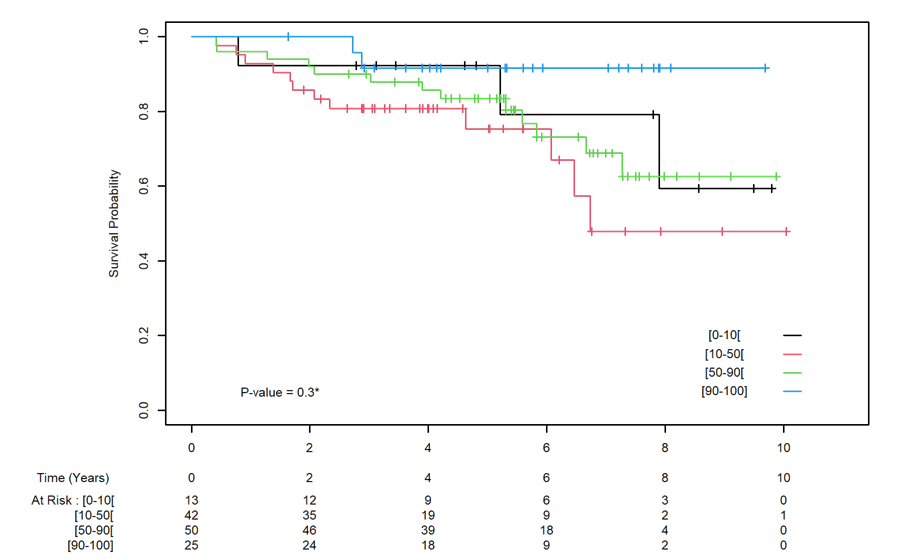


**Annex.1: Progression Free survival regarding to the percentage of capping**
